# Dexmedetomidine Can Enhance PINK1/Parkin-Mediated Mitophagy in MPTP-Induced PD Mice Model by Activating AMPK

**DOI:** 10.1155/2022/7511393

**Published:** 2022-04-12

**Authors:** Cheng Chen, Yaohua Chen, Tingting Liu, Dan Song, Di Ma, Oumei Cheng

**Affiliations:** ^1^Department of Neurology, The First Affiliated Hospital of Chongqing Medical University, Chongqing 400016, China; ^2^Laboratory Research Center, The First Affiliated Hospital of Chongqing Medical University, Chongqing 400016, China

## Abstract

Parkinson's disease (PD) is a common neurodegenerative disease characterized by the degeneration of dopaminergic (DA) neurons in the substantia nigra (SN). Our previous study has shown that dexmedetomidine (Dex) can protect mitochondrial function and reduce apoptosis in MPP^+^-induced SH-SY5Y cells. Evidences have shown that mitophagy is related to the development of PD. In this study, we investigated whether Dex can enhance mitophagy in MPTP-induced mice to play a neuroprotective effect. In our experiment, mice were injected with MPTP 30 mg/kg intraperitoneally for 5 consecutive days to establish a PD subacute model. Dex (30, 50, and 100 *μ*g/kg) was injected intraperitoneally 30 minutes before each injection of MPTP, respectively. Our results showed that Dex (50 *μ*g/kg) most significantly attenuated MPTP-induced motor dysfunction and restored TH-positive neurons in the SN, increased the expression of the antiapoptotic protein Bcl-2, and decreased the expression of apoptotic proteins cleaved casepase3, cleaved casepase9, and Bax. Moreover, Dex increased the activity of mitochondrial Complexes I-IV and decreased the level of oxidative stress, manifesting as decreased MDA levels and increased SOD and GSH-PX levels. Besides, under transmission electron microscopy, Dex increased the mitophagosome which is an autophagosome with a mitochondrion-like structure inside under the electron microscope. In addition, Dex could also increase the expression of mitophagy-related proteins p-AMPK, LC3II/I, PINK1, and Parkin and decrease P62. However, after using Compound C (CC, 10 mg/kg, AMPK inhibitor), the effects of Dex on increasing PINK1/Parkin-induced mitophagy and neuroprotection were attenuated. In conclusion, Dex may improve mitochondrial function by activating AMPK to enhance PINK1/Parkin-induced mitophagy, thereby protecting dopaminergic neurons.

## 1. Introduction

Parkinson's disease (PD) is a neurodegenerative disease characterized by the loss of dopaminergic neurons in the substantia nigra of the midbrain and the formation of Lewy bodies [1]. PD includes motor symptoms such as motor retardation, muscle rigidity, resting tremor, postural and gait instability, and a series of nonmotor symptoms [2]. In 2015, PD affected 6.2 million people worldwide and led to approximately 117,400 deaths [3]. However, the complex pathophysiological mechanisms of PD make the treatment of PD still a major problem at present. Deep brain stimulation (DBS) has been recognized as a clinically neurosurgical therapy for motor symptoms, which is a supplement to drug therapy [4, 5], and dexmedetomidine (Dex) is often used as the preferred general anesthetic in the treatment of PD by DBS [6].

Dex is a highly selective *α*2 adrenergic agonist with sedative, analgesic, anti-inflammatory, and antioxidant effects, which is currently commonly used in Parkinson's DBS surgery, and may have better efficacy and fewer side effects than clonidine and propofol, such as respiratory depression and dyskinesias [7–10]. Our previous studies have shown that Dex can protect MPP+-treated SH-SY5Y cells by alleviating the decline of the mitochondrial membrane potential [11]. Furthermore, it has been shown Dex could also improve the symptoms of 6-OHDA-induced Parkinson's rats by inhibiting inflammation [12], but the specific effect and mechanism of Dex on the MPTP-induced PD mice model are unclear.

Mitochondrial dysfunction is involved in the pathogenesis of PD [13, 14], which can cause oxidative stress, leading to apoptosis of dopaminergic neurons [15–17]. Mitophagy is an important part of the quality control of mitochondria [18] and can clear the damaged mitochondria to regulate mitochondrial function. Aberrant mitochondrial accumulation occurs in neuronal autophagosomes of PD patients [19–21]. Defective mitophagy flux and mitochondrial damage have also been demonstrated in PD models in vivo and in vitro [22, 23]. Therefore, enhancing mitophagy may be a potential strategy for the treatment of PD [24, 25].

The PINK1/Parkin pathway is a classic upstream pathway that regulates mitophagy [26], and enhancing PINK1/Parkin-mediated mitophagy can protect dopaminergic neurons in the MPP+/MPTP-induced PD model [23]. AMPK is a serine/threonine-protein kinase that can facilitate mitophagy through increasing fission and by promoting autophagosome initiation and lysosomal targeting [27]. AMPK*α*2 can enhance PINK1/Parkin-mediated mitophagy through PINK1 phosphorylation, thereby protecting cardiomyocytes in TAC-induced heart failure mice [28]. It has been reported that Dex can enhance PINK1/Parkin-mediated mitophagy to protect macrophages from inflammation and apoptosis in LPS-treated macrophages [29]. Furthermore, Dex pretreatment inhibited neuroinflammation via activation of AMPK in cerebral ischemia/reperfusion injury [30]. Dex could also protect against myocardium ischemic/reperfusion injury by activating an AMPK/PI3K/Akt/eNOS pathway in mice [31]. However, it has not been reported whether the effect of Dex on PD is achieved by regulating mitophagy. Thus, we hypothesize that Dex can enhance PINK1/Parkin-mediated mitophagy, improve mitochondrial function, reduce oxidative stress, and protect dopaminergic neurons by activating the expression of AMPK.

MPTP is a commonly used toxin to induce PD models, which is metabolized to the toxic cation MPP+ by monoamine oxidase-B enzymes, and then MPP+ interferes with the Complex I of the electron transport chain in the mitochondria, thereby damaging dopaminergic neurons [32]. In this study, we used MPTP-induced mice to explore the effect of Dex and its associated molecular mechanisms.

## 2. Experimental Materials and Methods

### 2.1. Experimental Animal

**Figure 1:**
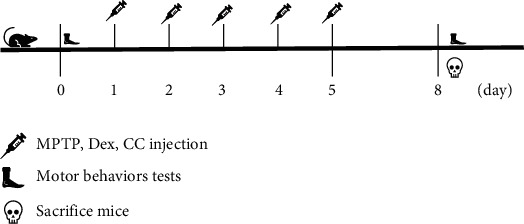
The specific process of mice receiving treatment.

Healthy 8-10-week-old C57 mice with a body weight of 18-22 g were obtained from the Experimental Animal Center of Chongqing Medical University. The animals were placed in a 12-hour light/dark cycle and had free access to water and food. All animal care and experiments were approved by the Ministry of Health of the People's Republic of China on the principles and guidelines for the care and use of laboratory animals and Chongqing Medical University. After one week of adaptation, the mice were randomly divided into 6 groups: the (1) control group, (2) Dex group, (3) CC group, (4) MPTP group, (5) MPTP+Dex group, and (6) MPTP+Dex+CC group. As shown in Figure 1, mice were injected with MPTP 30 mg/kg intraperitoneally for 5 consecutive days to establish a PD subacute model. Dex was injected intraperitoneally 30 minutes before MPTP injection [12, 33]. CC (10 *μ*g/kg) was injected intraperitoneally 30 minutes before Dex. The dose of CC was derived from the effect of CC on AMPK in previous studies [34].

### 2.2. Antibodies and Reagents

Dex (SML0956), MPTP (23007-85-4), and Compound C (866405-64-3) were purchased from the Sigma-Aldrich Chemical Co (St. Louis, USA). The primary antibodies anti-TH (ab137869), anti-LC3B (ab192890), anti-P62 (ab109012), anti-Parkin (ab77924), anti-PINK1 (ab216114), anti-AMPK (ab133448), anti-p-AMPK (ab134903), anti-cleaved caspase-3 (ab32042, activated form), and anti-cleaved caspase-9 (ab2324, activated form) were purchased from Abcam (Cambridge, MA, USA). Mfn2 (12186-1-AP), Drp1 (12957-1-AP), Bax (50599-2-Ig), Bcl-2 (26593-1-AP), GAPDH (6004-1-Ig), *β*-tubulin (10094-1-AP), goat anti-rabbit IgG (H+L), rhodamine conjugate (TRITC, SA00007-2), goat anti-rabbit IgG (H+L), FITC conjugate (FITC, SA00003-2), and monoclonal antibody (4B2E10) were obtained from ProteinTech (Wuhan, China).

### 2.3. Behavioral Test

#### 2.3.1. Open Field Experiment

Before the experiment, the mice were placed in the environment to adapt for 1 hour. During the experiment, the mice were placed in the center of the open field box (50 cm × 50 cm), and the total distance of the mice in the open field box was observed for 5 minutes.

#### 2.3.2. Pole Climbing Experiment

Take a rod with a length of 50 cm and a diameter of 2 cm, install a small wooden ball on the top of the rod, and cover it with gauze to prevent the mice from slipping. After the mice have undergone adaptation training, the time required for the mice to turn completely downward (T-turn) and climb down to the floor (T-LA) was recorded. Each mouse was tested 3 times, and the average time was calculated. If the mouse stopped halfway or climbed backwards, the test was repeated.

#### 2.3.3. Roller Experiment

The SD-2 roller instrument was used to measure the roller behavior of mice. The specific method is to train the mice continuously for 3 days before the test, and the mice with uncoordinated movements were eliminated. During the test, the mice were placed in the roller machine, the rotation speed was set to 35 r/min, the test time was 120 s, and the time for the mouse to start rotating from the roller to leaving the roller within 120 s was measured. Each mouse was measured 3 times, and the average value was taken.

### 2.4. Tissue and Slice Preparation

After the behavioral test, the mice were anesthetized with sodium pentobarbital, and the left ventricle was perfused with PBS and 4% paraformaldehyde. According to the anatomical position, the mouse midbrain area was taken out and frozen in liquid nitrogen for subsequent experiments. In each group, we take 5 mouse brain samples, place them in 4% paraformaldehyde at 4°C overnight, and transfer them to 20% and 30% sucrose solutions for overnight dehydration. Then, the tissues were embedded w4ith OCT and placed in a refrigerator at −80°C, and a cryostat (Leica Microsystems) was used for coronal sectioning (thickness 6-8 *μ*m).

### 2.5. Immunofluorescence

The brain slices were taken out from -80°C, rewarmed in a 37°C incubator for one hour, ruptured with 0.3% Triton, and blocked with goat serum for 1 hour, and then the slides were incubated with primary antibodies against TH, PINK1, and Parkin at 4°C overnight. After removing the primary antibody, the fluorochrome-conjugated secondary antibody was added and incubated for 1 hour, and then a few drops of DAPI were added. The excitation wavelength and the emission wavelength of the rhodamine conjugate (TRITC, SA00007-2) are 550 nm and 570 nm, respectively. The excitation wavelength and the emission wavelength of the FITC conjugate (FITC, SA00003-2) are 492 nm and 520 nm, respectively. Next, the slices were observed under a fluorescence microscope (DMi8 type, Leica, Germany). Calculate the fluorescence intensity. All images are processed by the ImageJ software (NIH, USA).

### 2.6. Detection of Mitochondrial Function

The activities of the mitochondrial Complexes I-IV were determined by the Micro Mitochondrial Respiratory Chain Complex I-IV Activity Assay Kit (Solarbio, China) according to the manufacturer's instructions. Briefly, mitochondrial homogenates were added into the respective reaction buffer. The reaction mixture was transferred to a prewarmed (30°C) quartz cuvette and immediately put into a spectrophotometer. The absorbance of the reaction mixture was measured at 340 nm for Complex I, 605 nm for Complex II, and 550 nm for Complexes III and IV, respectively. The mitochondrial complex activity was expressed as nmol/min/mg protein.

### 2.7. Determination of ROS, MDA, SOD, and GSH-PX

The concentration of MDA, SOD, and GSH-PX in mouse tissue homogenate was tested in accordance with the manufacturer's instructions (Nanjing Jiancheng Institute of Biological Engineering).

### 2.8. Western Blot

The midbrain tissue previously stored in liquid nitrogen was taken out and lysed on ice, then dissolved in RIPA buffer containing a mixture of protease and phosphatase inhibitors for 30 minutes. The lysate was centrifuged, and the supernatant was collected. The protein concentration was determined by the BCA method. The protein (25-30 *μ*g) was loaded onto each lane. Then, we used the 10-16% SDS-PAGE gel electrophoresis to transfer the separated proteins to the PVDF membrane, and they were blocked with 5% skim milk for 1 hour and were incubated with primary antibodies at 4°C overnight. The PVDF membranes were incubated with the corresponding secondary antibody at room temperature for one hour and washed with TBST for 10 min × 3 times. The gray value of the target protein and the gray value of the internal reference were used for semiquantitative analysis, and the ratio of the gray value of phosphorylated protein kinase to the gray value of the respective target total protein kinase was used to indicate the degree of phosphorylation.

### 2.9. Transmission Electron Microscopy (TEM)

For TEM, the midbrain tissues were fixed in 2.5% glutaraldehyde for 4 h at 4°C. Then, the pellets were postfixed in 1% osmium tetroxide/0.1 M phosphate buffer (pH = 7.4) and dehydrated serial dilutions in acetone and embedded with the SPI-PON 812 Epoxy Resin Monomer (SPI Supplies Division, Structure Probe Inc., West Chester, PA). Ultrathin sections (60–80 nm) were stained with uranyl acetate and lead citrate and observed with TEM (Hitachi, Tokyo, Japan).

### 2.10. Statistical Analysis

The statistical results were calculated using the GraphPad 8.0 and SPSS 20.0 statistical software, and all data were expressed as mean ± standard error. Data comparison between groups was done using the single-factor analysis of variance combined with Turkey's multiple comparisons. *P* < 0.05 is considered statistically different.

## 3. Results

### 3.1. Dex Improved Motor Symptoms in MPTP-Induced PD Mice

**Figure 2:**
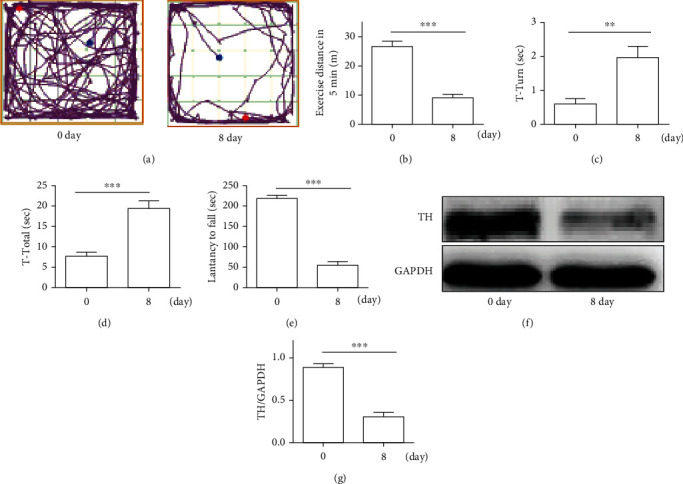
MPTP caused motor dysfunction in mice and the decrease of TH. (a, b) Exercise distance in 5 min. (c) T-turn time in the pole test. (d) T-total time in the pole test. (e) Latency to fall. (f) The expression of TH was analyzed by western blot. (g) TH/GAPDH. Values are represented in the form of mean ± SD. (*n* = 10). ^∗^*P* < 0.05, ^∗∗^*P* < 0.01, and ^∗∗∗^*P* < 0.001, ns: not significant.

**Figure 3:**
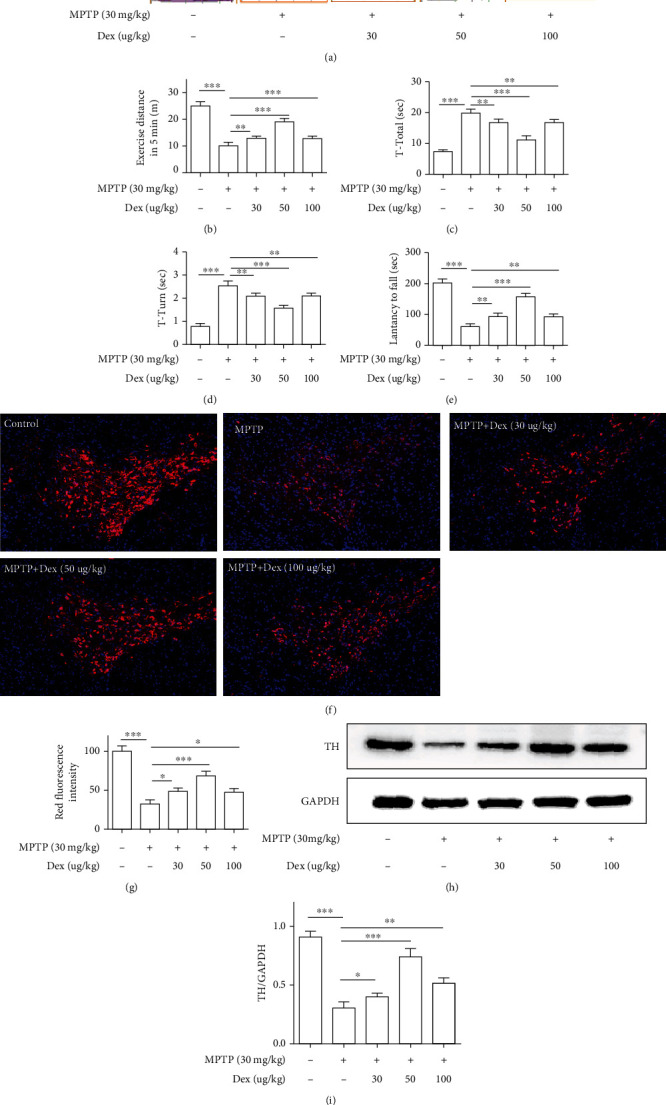
Dex relieved MPTP-induced motor dysfunction in mice and increased the expression of TH-positive neurons. (a, b) Exercise distance in 5 min. (c) T-turn time in the pole test. (d) T-total time in the pole test. (e) Latency to fall. (f) Representative immunofluorescence images. (g) The statistical results of TH fluorescence intensity in the SNpc area of each group. (h) Relative expression of TH in SN of mice was studied using the western blot technique. (i) Densitometry analysis of proteins. Values are represented in the form of mean ± SD. (*n* = 10). ^∗^*P* < 0.05, ^∗∗^*P* < 0.01, and ^∗∗∗^*P* < 0.001, ns: not significant.

We tested the mice's motor symptoms on the 0th day (that is no MPTP injection) and the 8th day (that is the third day after 5 consecutive injections of MPTP). As shown in Figure 2, compared with the 0th day, mice on the 8th day reduced the distance of movement in the field experiment, increased the pole climbing time, and significantly decreased the residence time in the roller experiment (Figures 2(a)–2(e); *P* < 0.01). On the 8th day, western blot results indicated that MPTP caused the loss of dopamine neurons (Figures 2(f) and 2(g); *P* < 0.001), so we chose the 8th day as the follow-up observation time and our model. As shown in Figure 3, in comparison with the MTPP group, the mice in the MPTP+Dex (30 *μ*g/kg) group, the MPTP+Dex (50 *μ*g/kg) group, and the MPTP+Dex (100 *μ*g/kg) increased the exercise distance, but the increase in the MPTP+Dex (50 *μ*g/kg) group was more obvious (Figures 3(a) and 3(b); *P* < 0.001), and there was no significant difference between the MPTP+Dex (30 *μ*g/kg) group and the MPTP+Dex (100 *μ*g/kg) group. Compared with the MPTP group, the mice in the MPTP+Dex (30 *μ*g/kg) group, MPTP+Dex (50 *μ*g/kg) group, and MPTP+Dex (100 *μ*g/kg) group had relatively less head-turning time and total pole climbing time, and the MPTP+Dex (50 *μ*g/kg) group had the most time reduction (Figures 3(c) and 3(d); *P* < 0.001). Similarly, in the rotating rod experiment, compared with the MPTP group, in the MPTP+Dex (30 *μ*g/kg) group, MPTP+Dex (50 *μ*g/kg) group, and MPTP+Dex(100 *μ*g/kg) group, the time of mice stay on the rod increased, and the MPTP+Dex (50 *μ*g/kg) group increased the most (Figure 3(e); *P* < 0.001), but there was no significant difference between the MPTP+Dex (30 *μ*g/kg) group and MPTP+Dex (100 *μ*g/kg) group. Our results showed that Dex improved the motor symptoms of Parkinson's mice, and the concentration of 50 *μ*g/kg has the most obvious effects.

### 3.2. Dex Rescued the Loss of TH-Positive Neurons in MPTP-Induced PD Mice

The loss of dopaminergic neurons in the substantia nigra is the main pathological feature of PD. We used immunofluorescence to detect TH-labeled dopamine neurons in the substantia nigra of mice. As shown in Figures 3(f) and 3(g), the immunofluorescence results showed that compared with the control group, the TH+ dopaminergic neurons in the MPTP group were significantly reduced (*P* < 0.001), Dex (30, 50, and 100 *μ*g/kg) pretreatment can protect dopaminergic neurons from MPTP damage to a certain extent (*P* < 0.01), and the 50 *μ*g/kg effect is the most obvious. In addition, we quantified the expression of TH-positive neurons by western blot; consistent with the immunofluorescence results, Dex (50 *μ*g/kg) reduced MPTP damage to TH-positive neurons most obviously (Figures 3(h) and 3(i); *P* < 0.001). Therefore, combining the behavioral and pathological results, we chose the 50 *μ*g/kg concentration as the subsequent experiment concentration.

### 3.3. Dex Reduced Oxidative Stress and Reduced Dopaminergic Neuron Apoptosis

**Figure 4:**
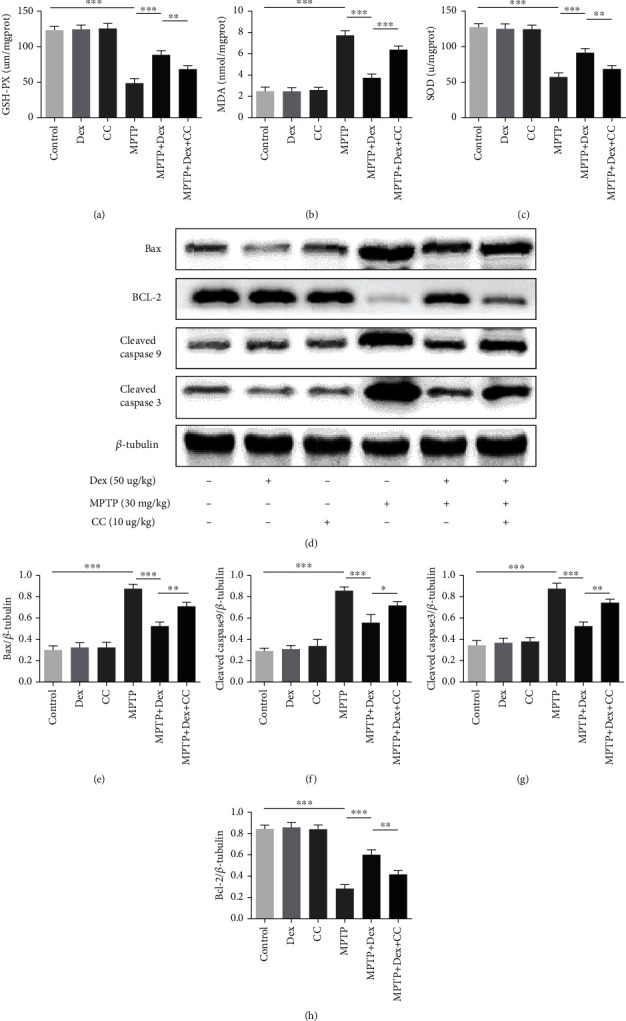
The effect of Dex on oxidative stress and apoptosis. (a–c) The level of oxidative stress. (d) The relative expression of cleaved caspase-3, cleaved caspase-9, Bax, and antiapoptotic protein Bcl-2 in SN of mice was studied using the western blot technique. (g, h) Densitometry analysis of proteins. Values are represented in the form of mean ± SD. (*n* = 5). ^∗^*P* < 0.05, ^∗∗^*P* < 0.01, and ^∗∗∗^*P* < 0.001, ns: not significant.

In order to explore the effects of Dex on oxidative stress in Parkinsonian mice, we tested the levels of GSH-PX, SOD, and MDA in the substantia nigra. Our results showed that there was no statistical difference in GSH-PX, SOD, and MDA between the control group, Dex group, and CC group. Compared with the control group, the GSH-PX and SOD in the tissues of the MPTP group decreased (Figures 4(a) and 4(c); *P* < 0.001), and the MDA increased (Figures 4(b) and 4(g); *P* < 0.001). After Dex pretreatment, the levels of antioxidant enzymes SOD and GSH-PX increased, and the oxidation product MDA decreased (Figures 4(a)–4(c); *P* < 0.001), while CC weakened these above effects of Dex (Figures 4(a)–4(c); *P* < 0.01). Oxidative stress can lead to neuronal deformation and apoptosis. We further detected the levels of apoptosis proteins cleaved caspase-3, cleaved caspase-9, and Bax and antiapoptotic protein Bcl-2. Compared with the control group, the levels of cleaved caspase-3, cleaved caspase-9, and Bax in the MPTP group were increased (Figures 4(d)–4(g); *P* < 0.001), while the level of Bcl-2 was decreased (Figures 4(d) and 4(h); *P* < 0.001). After Dex pretreatment, the apoptotic proteins cleaved caspase-3, cleaved caspase-9, and Bax decreased (Figures 4(d)–4(g); *P* < 0.001), the antiapoptotic protein Bcl-2 increased (Figures 4(d) and 4(h); *P* < 0.001) compared with the MPTP group, and CC decreased these above effects of Dex (Figures 4(a)–4(h); *P* < 0.01). Our results suggested that Dex could reduce the level of oxidative stress and apoptosis in Parkinson's mice.

### 3.4. Dex Regulated Mitochondrial Function in MPTP-Induced PD Mice

**Figure 5:**
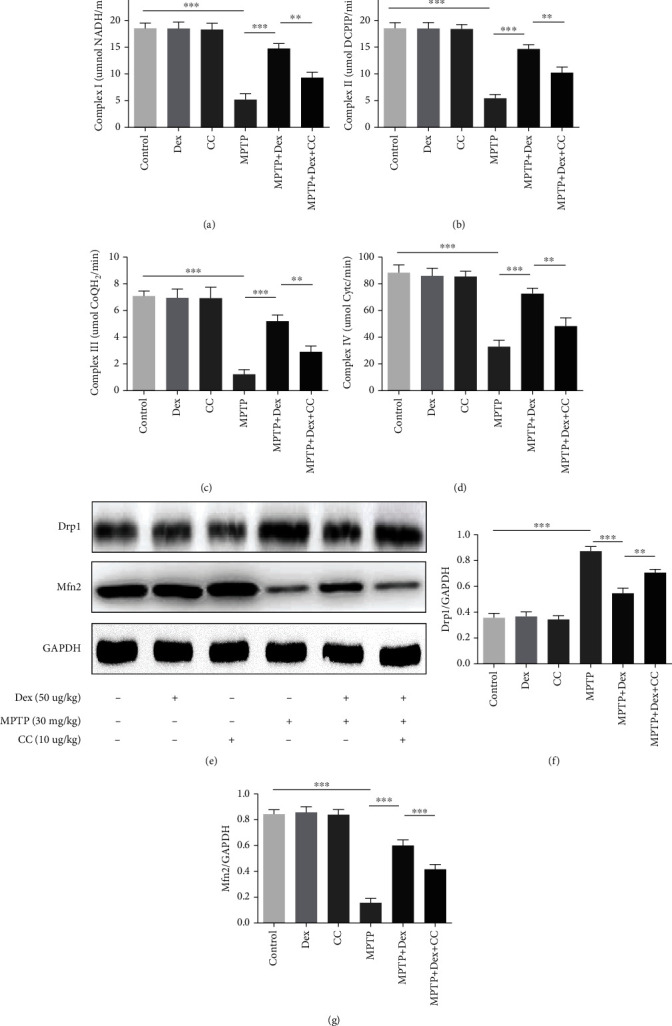
The effect of Dex on mitochondrial function. (a–d) Dex-mediated alterations on mitochondrial Complexes I-IV. (e) Relative expression of Mfn2 and Drp1 in SN of mice was studied using the western blot technique. (g, f) Densitometry analysis of proteins. Values are represented in the form of mean ± SD. (*n* = 5). ^∗^*P* < 0.05, ^∗∗^*P* < 0.01, and ^∗∗∗^*P* < 0.001, ns: not significant.

Mitochondrial dysfunction is one of the pathogenic factors of PD. There are many ways to detect mitochondrial function, such as the activity of mitochondrial Complexes I-IV and mitochondrial division fusion protein (Drp1, Mfn2). Our results showed that there were no significant differences in the mitochondrial Complex I-IV activity and the expression levels of Drp1 and Mfn2 in the control group, Dex group, and CC group. Compared with the control group, in the MPTP group, the activity of mitochondrial Complexes I-IV was decreased (Figures 5(a)–5(d); *P* < 0.001), and the expression level of Mfn2 decreased (Figures 5(e) and 5(g); *P* < 0.001), while the expression level of Drp1 increased compared with the control group (Figures 5(e) and 5(f); *P* < 0.001). In comparison with the MPTP group, in the Dex+MPTP group, the activity of mitochondrial Complexes I-IV was increased (Figures 5(a)–5(d); *P* < 0.001), and the expression level of Mfn2 was increased (Figures 5(e) and 5(g); *P* < 0.0014), while the expression level of Drp1 decreased (Figures 5(e) and 5(f); *P* < 0.001). After adding the AMPK inhibitor CC, these effects of Dex were weakened (Figures 5(a)–5(g); *P* < 0.01). Our results showed that Dex can save the mitochondrial function in Parkinson's mice.

### 3.5. Dex Enhances Mitophagy

**Figure 6:**
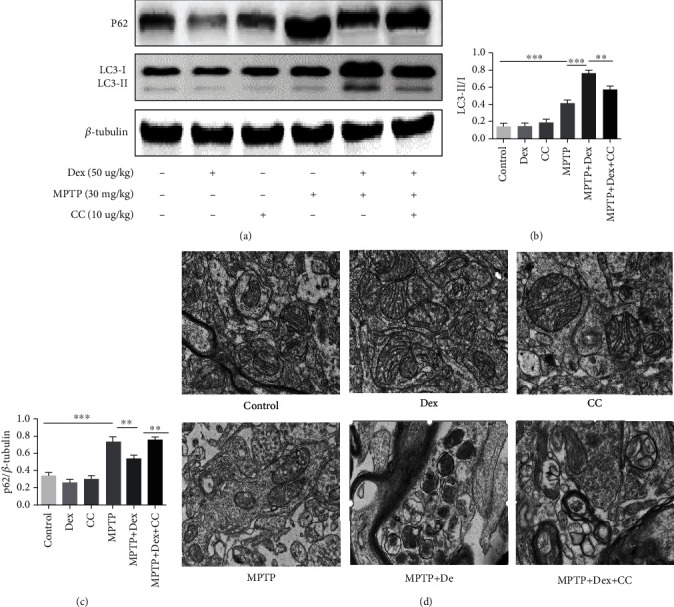
The effect of Dex on mitophagy. (a) Representative TEM image of mitophagy autophagosomes in the SN of mice. (b) The level of LC3 and P62 was analyzed by western blot. (c, d) Densitometric quantification of LC3 and P62. Values are represented in the form of mean ± SD. (*n* = 5). ^∗^*P* < 0.05, ^∗∗^*P* < 0.01, and ^∗∗∗^*P* < 0.001, ns: not significant.

Upregulation of LC3II/LC3I and downregulation of p62 at the same time are biomarkers of autophagic flux [35]. Therefore, we detected the autophagy-related proteins LC3B and P62. Our results showed that there was no statistically significant difference in the expression of LC3II/I and P62 in the control group, Dex group, and CC group. Compared with the control group, the expression of LC3II/I and P62 in the MPTP group increased (Figures 6(a)–6(c); *P* < 0.001). At this time, the lysosome has not been able to bind to the autophagosome, that is, the autophagic flow is obstructed. However, in the Dex+MPTP group, LC3II/I further increased, but P62 decreased compared with the MPTP group (Figures 6(a)–6(c); *P* < 0.001), which indicated that autophagosomes can be effectively degraded by lysosomes at this time and autophagic flux increased. Then we used TEM to observe whether mitophagy occurred. In our experiment, the results of TEM showed that in the MPTP group, the mitochondria swelled, and the mitochondrial cristae disappeared and had less autophagosomes and mitophagosome. Compared with the MPTP group, the Dex+MPTP group can protect the mitochondrial structure and increase mitophagosomes which is an autophagosome with a mitochondrion-like structure inside (Figure 6(d)).

### 3.6. Dex Enhances PINK1/Parkin-Mediated Mitophagy by Activating AMPK

**Figure 7:**
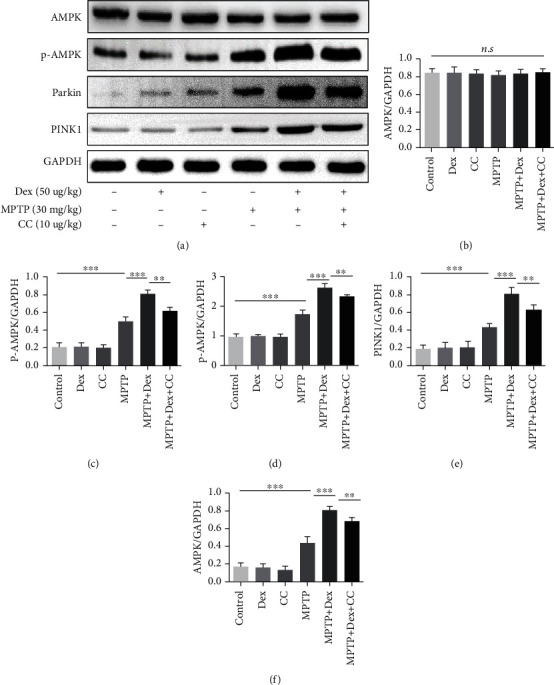
Dex regulated the expression of p-AMPK, PINK1, and Parkin. (a) The expression of AMPK, p-AMPK, PINK1, and Parkin was analyzed by western blot. (b–f) Densitometric quantification of AMPK, p-AMPK, PINK1, and Parkin. Values are represented in the form of mean ± SD. (*n* = 5). ^∗^*P* < 0.05, ^∗∗^*P* < 0.01, and ^∗∗∗^*P* < 0.001, ns: not significant.

**Figure 8:**
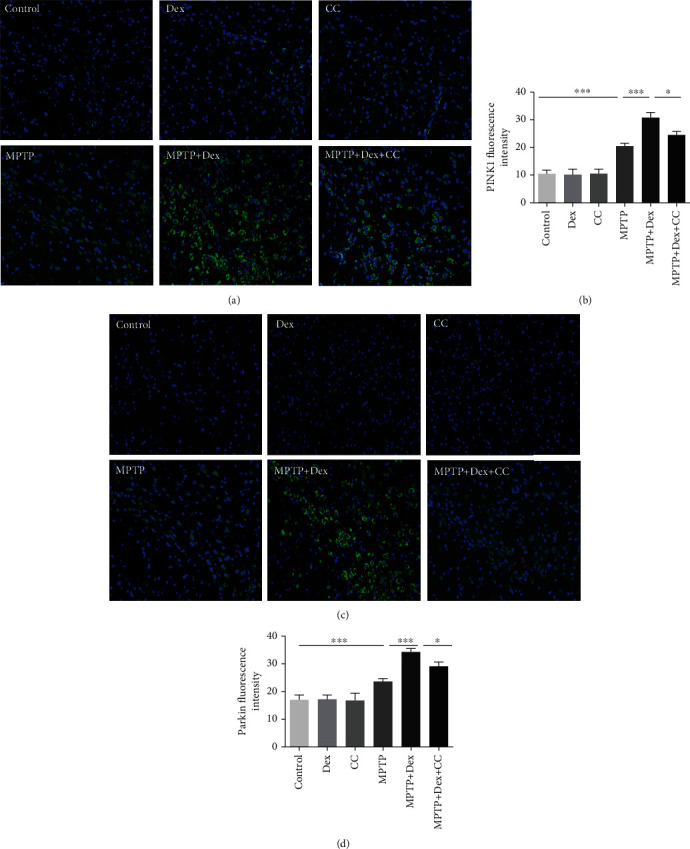
The effect of Dex on PINK1- and Parkin-labeled neurons. (a, b) Representative immunofluorescence images of PINK1 and Parkin in SN of mice. (c, d) The relative fluorescence intensity of PINK1 and Parkin. Values are represented in the form of mean ± SD. (*n* = 5). ^∗^*P* < 0.05, ^∗∗^*P* < 0.01, and ^∗∗∗^*P* < 0.001, ns: not significant.

AMPK has been shown to be related to mitophagy [36, 37]. Our results showed that after MPTP treatment, the expression of p-AMPK increased (Figures 7(a) and 7(c); *P* < 0.001), the ratio of p-AMPK/AMPK increased (Figures 7(a) and 7(d); *P* < 0.001), and the expression of PINK1 and Parkin proteins increased in comparison with the control group (Figures 7(a), 7(e), and 7(f); *P* < 0.001). After Dex pretreatment, the ratio of p-AMPK/AMPK and the expression of PINK1 and Parkin increased further compared with the MPTP group (Figures 7(a) and 7(d)–7(f); *P* < 0.001). But among the 6 groups, there was no difference in AMPK (Figures 7(a) and 7(b)). However, when CC was used to inhibit AMPK, the expression of PINK1 and Parkin also decreased correspondingly with the decrease of p-AMPK compared with the Dex+MPTP group (Figures 7(a) and 7(c)–7(f); *P* < 0.001). Similarly, as shown in Figure 8, compared with the control group, the fluorescence intensity of PINK1 and Parkin-positive neurons in the MPTP group increased significantly (*P* < 0.001). Pretreatment with Dex can increase the fluorescence intensity of PINK1 and Parkin-positive neurons further compared with the MPTP group (*P* < 0.001). However, compared with the MPTP+Dex group, pretreatment with CC decreased the mitophagy flux and the expression of p-AMPK. In short, our results proved that Dex can increase the PINK1/Parkin-induced mitophagy by activating AMPK, and the AMPK inhibitors of CC reversed the effect of Dex.

## 4. Discussion

This study proved that Dex could protect dopaminergic neurons through PINK1/Parkin-mediated mitophagy in MPTP-induced mice by activating AMPK. The evidence is as follows: (1) Dex relieved Parkinson's motor symptoms and rescued the loss of TH+ neurons induced by MPTP. (2) Dex enhanced PINK1/Parkin-mediated mitophagy to protect mitochondrial function, which could reduce oxidative stress and apoptosis of dopaminergic neurons in SN. (3) The effects of Dex on PINK1/Parkin-mediated mitophagy were achieved by activating AMPK.

Dex is a highly selective *α*2 adrenergic agonist with sedative, analgesic, anti-inflammatory, and antioxidant effects [38]. In 6-OHDA-induced rats, Dex could improve motor symptoms, inhibit inflammation, and increase the level of DA in the striatum [12]. In our study, we found that after 5 days of continuous intraperitoneal injection of MPTP 30 mg/kg, which is consistent with previous models of PD [39], the behavioral disorders were most obvious on the 3rd day, so we chose the 3rd day as the time point for subsequent observation of various indicators. We found that Dex pretreatment could significantly improve movement disorders in PD which is also consistent with previous research [12]. However, 50 *μ*g/kg Dex pretreatment was more effective than 30 *μ*g/kg and 100 *μ*g/kg, suggesting that the effect of Dex is not dose-dependent. A study has reported that intraperitoneal injection of Dex (300 *μ*g/kg) can increase the phosphorylation and accumulation of tau in the hippocampus of C57 mice and may affect spatial reference memory [40]. In addition, previous research has also demonstrated that the effect of a high concentration of Dex is not necessarily more noticeable [41], which is consistent with our study. So, the above several pieces of studies and our research both prove that the effect of Dex is not dose-dependent.

The mitochondria are organelles with a double-layer membrane structure. When the mitochondria are damaged, their morphology and structure change, such as mitochondria swelling, rupture, and mitochondrial cristae abnormalities [42, 43]. The activity of the mitochondrial respiratory chain can reflect mitochondrial function. The mitochondrial respiratory chain is located on the inner mitochondrial membrane and consists of 5 complexes (Complexes I-V) [44]. Besides, in order to adapt to changes in the cellular environment and maintain mitochondrial functions, the mitochondria continue to undergo the process of division and fusion, collectively referred to as “mitochondrial dynamics” [45]. Mitochondrial fusion protein 2 (mitofusin 2 (Mfn2)) is the major protein to mediate mitochondrial fusion in mammalian cells. The proteins that mediate mitochondrial division mainly include mitochondrial motility-related protein 1 (dynamin-related protein 1 (Drp1)). A previous study has shown that Mfn2 was decreased and Drp1 was increased when mitochondrial dysfunction occurs [46]. Consistent with the previous results, our study found that the MPTP decreased the activity of mitochondrial Complexes I-V and Mfn2 and increased the expression of Drp1 when compared with the control group [47–49]. Pretreatment with Dex had the opposite result in MPTP-induced mice, which indicates that Dex can improve mitochondrial function.

The mitochondria are the main site of oxidative stress [18, 50, 51]. Damaged mitochondria will increase the level of oxidative stress, which can cause further damage to the mitochondria [52, 53]. Oxidative stress and mitochondrial dysfunction are the two most important causes of PD [54–56]. Under normal circumstances, the body's antioxidant system and oxidation system maintain a dynamic balance. While under various harmful stimuli such as toxins, the cell's antioxidant substances such as glutathione peroxidase (GSH) and superoxide dismutase (SOD) cannot remove harmful substances such as ROS in time leading to the occurrence of oxidative stress which can easily to damage the substantia nigra area [57]. When oxidative damage occurs, it may cause lipid peroxidation (LPO) and malondialdehyde (MDA) which is an index of LPO. Mitochondrial dysfunction can cause oxidative stress, resulting in the apoptosis of dopaminergic neurons [15]. Chlorogenic acid can increase peroxidase and glutathione peroxidase in PD and prevent the MPTP-mediated apoptotic cascade reaction [48]. Our results showed that the Dex group decreased MDA and increased GSH and SOD compared with the MPTP group, which is consistent with the previous study [58]. Excessive oxidative stress can contribute to the mitochondrial apoptotic pathways in cells [59]. In this pathway, the Bcl-2 family protein Bax relocates to the surface of mitochondria, resulting in a decrease in membrane potential and an increase in membrane permeability, and then the proapoptotic factor cytochrome c (CytC) in the mitochondria is released into the cytoplasm to activate caspase-9, which further activates caspase-3, thereby initiating the caspase cascade reaction, which eventually leads to cell apoptosis [60]. Our research found that Dex can decrease the expression of apoptotic proteins cleaved caspase-3, cleaved caspase-9, and Bax and increase the expression of antiapoptotic protein Bcl-2. These results indicate that Dex can protect mitochondrial function, reduces oxidative stress, and reduces apoptosis.

Mitophagy is an important factor regulating mitochondrial function, and enhancing mitophagy is an effective way to enhance mitochondrial function [61]. In PD, growing evidence suggested that impaired mitophagy are important mechanisms for the development of the disease [62], which can clear the damaged mitochondria to regulate mitochondrial function [26]. The PINK1/Parkin pathway is a classic upstream pathway that regulates mitophagy. The expression of mitophagy-related genes PRKN, PINK1, and DJ-1 can be observed in PD patient cells [63]. Studies have shown that mitochondria accumulate abnormally in the autophagosomes of neurons in PD patients [19–21]. When mitochondria are damaged, the mitochondrial inner membrane potential decreases, and PINK1 can be stably expressed on the outer mitochondrial membrane, and then Parkin in the cytoplasm is shifted to the mitochondria through its phosphorylation and activated. Finally, the protein p62 binds to LC3 to form a mitophagosome, which is then degraded by lysosomes [64–67], thereby eliminating impaired mitochondria. And according to reports, enhancing the PINK1/Parkin-mediated mitophagy can protect the dopaminergic neurons in MPP+/MPTP-induced PD models [24]. In our experiment, we found that Dex increased autophagic flux, which was manifested as an increase in LC3II/I and a decrease in P62. Under TEM, we observed that the mitochondria swelled, and the mitochondrial cristae disappeared, but there were fewer autophagosomes and mitophagosome in the MPTP group. Dex increased the mitophagosome which is an autophagosome with a mitochondrion-like structure inside. In order to further support the results of TEM, we next explored the classic PINK1/Parkin pathway of mitophagy. After MPTP treatment, western blot and immunofluorescence results showed that the expression of PINK1 and Parkin increased, and Dex further increased the expression of PINK1 and Parkin. Our results all implied that autophagosomes enveloped mitochondria, but mitophagosome failed to be degraded by lysosomes; therefore, mitophagy flux was impaired after MPTP treatment, which is consistent with the results of the previous study [68]. However, after Dex pretreatment, PINK1, Parkin, and the ratio of LC3II/I were increased with the decreased P62, which indicated that mitophagy flux increases and that impaired mitochondria were eliminated. At this time, mitophagy could partially protect neurons from MPTP damage. In addition, Dex alone had no effect on mitophagy. Therefore, our results showed that Dex could enhance PINK1/Parkin-mediate mitophagy in MPTP-induced mice.

AMPK is a serine/threonine-protein kinase that exists in eukaryotic cells. It is an energy sensor that can regulate the energy metabolism of the body and cells and is considered a protector of mitochondria [69]. According to reports, in the MPTP-induced PD model, the activation of AMPK can prevent neuronal cell death [70]. In addition, AMPK can activate autophagy [71]. Recent studies have also found that the AMPK signaling pathway is related to the process of mitophagy [36, 37]. In TAC-induced heart failure mice, AMPK*α*2 can enhance PINK1/Parkin-mediated mitophagy to protect cardiomyocytes through PINK1 phosphorylation [28]. Growing evidences have reported that Dex can promote the expression of AMPK [30]. Therefore, we further study the role of AMPK in the protective effect of Dex on the MPTP-induced PD model. In our experiment, we found that Dex could promote the expression of p-AMPK which is the activated form of AMPK, but the expression level of AMPK did not change, which means that Dex could activate AMPK. After pretreatment with the AMPK inhibitor CC, the effects of Dex on enhancing mitophagy and reducing oxidative stress and reducing neuronal apoptosis were reversed. Our results suggest that CC abolished the neuroprotective effect of Dex, and the protective effect of Dex may be exerted by activating AMPK to upregulate mitophagy. We also noticed that after MPTP treatment, p-AMPK also elevated, which may be a self-protection measure for neurons, but at this time, the mitophagy flux was impaired, implying that this response cannot clear the damaged mitochondria in time. Therefore, the increase in p-AMPK at this time may compensate for cytotoxicity, but the cells maintain their self-regulation for a short time and eventually undergo apoptosis, which is consistent with the results of a previous study [68].

## 5. Conclusion

This study demonstrated the neuroprotective effects of Dex on the MPTP-induced mice. Dex can activate AMPK-dependent PINK1/Parkin-mediated mitochondrial autophagy, rescue the mitochondrial dysfunction of Parkinsonian mice, reduce oxidative stress, and reduce neuronal apoptosis. Dex may be used as a potential drug for the treatment of Parkinson's disease, and our results also provide theoretical support for the use of Dex in Parkinson's disease DBS surgery.

## Data Availability

The data used to support the findings of this study are available from the corresponding author upon request.

## Abbreviations

Dex: Dexmedetomidine
CC: Compound C
PD: Parkinson's disease
DBS: Deep brain stimulation
SN: Substantia nigra
MPTP: 1-Methyl-4-phenyl-1,2,3,6-tetrahydropyridine
MPP+: 1-Methyl-4-phenylpyridinium
ROS: Reactive oxygen species
MMP: Mitochondrial membrane potential
